# Phage Therapy for Multi-Drug Resistant Respiratory Tract Infections

**DOI:** 10.3390/v13091809

**Published:** 2021-09-11

**Authors:** Joshua J. Iszatt, Alexander N. Larcombe, Hak-Kim Chan, Stephen M. Stick, Luke W. Garratt, Anthony Kicic

**Affiliations:** 1 Occupation, Environment and Safety, School of Population Health, Curtin University, Perth 6845, Australia; joshua.iszatt@telethonkids.org.au (J.J.I.); alexander.larcombe@telethonkids.org.au (A.N.L.); 2 Wal-yan Respiratory Research Centre, Telethon Kids Institute, Perth 6009, Australia; stephen.stick@telethonkids.org.au (S.M.S.); luke.garratt@telethonkids.org.au (L.W.G.); 3 Advanced Drug Delivery Group, Sydney Pharmacy School, University of Sydney, Camperdown 2006, Australia; kim.chan@sydney.edu.au; 4 Department of Respiratory and Sleep Medicine, Perth Children’s Hospital, Perth 6009, Australia; 5 Centre for Cell Therapy and Regenerative Medicine, School of Medicine and Pharmacology, Harry Perkins Institute of Medical Research, The University of Western Australia, Nedlands 6009, Australia

**Keywords:** multi-drug resistance, bacteriophage, respiratory, infectious disease

## Abstract

The emergence of multi-drug resistant (MDR) bacteria is recognised today as one of the greatest challenges to public health. As traditional antimicrobials are becoming ineffective and research into new antibiotics is diminishing, a number of alternative treatments for MDR bacteria have been receiving greater attention. Bacteriophage therapies are being revisited and present a promising opportunity to reduce the burden of bacterial infection in this post-antibiotic era. This review focuses on the current evidence supporting bacteriophage therapy against prevalent or emerging multi-drug resistant bacterial pathogens in respiratory medicine and the challenges ahead in preclinical data generation. Starting with efforts to improve delivery of bacteriophages to the lung surface, the current developments in animal models for relevant efficacy data on respiratory infections are discussed before finishing with a summary of findings from the select human trials performed to date.

## 1. Introduction

Any form of immunocompromised phenotype, whether from a muco-obstructive disorder, organ transplant, or chemotherapy, increases the chances of acquiring bacterial respiratory tract infections [1]. Unlike acute infections, persistent lung infections can extend to months or longer and are not so easily eradicated using standard antibiotic treatments, often due to resistance mechanisms employed by pathogens [2]. The ability of several opportunistic bacterial species to continually gain antibiotic resistance mechanisms and become multi-drug resistant (MDR) has stifled the treatment of many communicable diseases in recent years [3].

**Figure 1:**
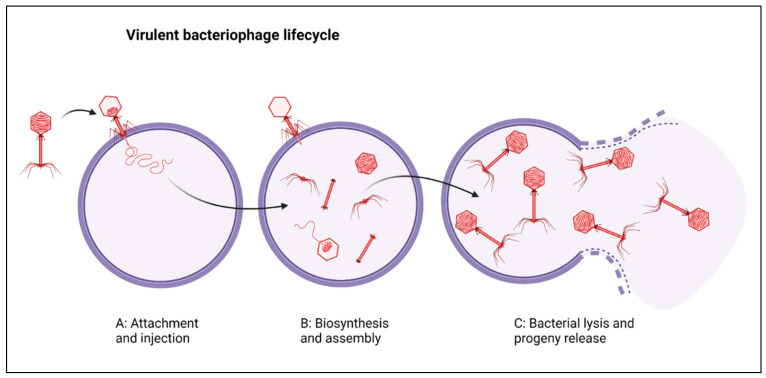
(**A**) Phage (depicted in red) attachment and injection of its genome into a host bacterium (circular purple cell). (**B**) Bacterial cellular machinery is used to synthesise and assemble new phage progeny. (**C**) Progeny are released due to bacterial cell lysis. Created with BioRender.com (31 May 2021).

Lower respiratory tract infections (LRTIs) are estimated to have caused approximately 2.74 million deaths in 2015 across 195 countries [4]. In addition to mortality, LRTIs cause severe decreases in respiratory quality of life, estimated at 103 million LRTI disability-adjusted life-years [4,5]. Recent estimates suggest that the median number of attributable deaths caused by MDR respiratory infections has more than doubled from 2007 to 2015 [6]. This rise in antibacterial resistance has occurred in parallel with dramatic decreases in the number of promising antibiotic pipelines, leading to a renewed interest in phage, viruses that can infect and kill bacteria [7,8]. Phage therapy (PT) entails the administration of virulent bacteriophage (phage) to treat bacterial infections and dates back to the early 1900s, when it was considered a potential alternative treatment to serum injections and used by Felix d’Herelle, Richard Bruynoghe, and Joseph Maisin to treat bacterial dysentery and staphylococcal skin diseases [9,10,11]. PT typically involves high titres (>10^9^) of virulent and adequately characterised phage to ensure clearance of the bacterial infections [12]. These phages infect, replicate within, and lyse bacterial cells in order to release phage progeny (Figure 1) and this process results in a rapid decrease in specific bacterial populations.

Whilst PT has been predominantly directed towards bacterial pathogens, recent progress in the understanding of phage immunobiology has renewed interest in PT repurposed to indirectly target viral infections [13,14,15,16]. In the context of this review, PT will refer to the use of phage to target bacterial infections with a focus on pulmonary bacterial pathogens. Phages have several clinically relevant advantages over antibiotics and, more importantly, are able to rapidly reduce bacterial populations in situations of antibiotic resistance [12,15,16]. In lieu of increasing antibacterial resistance, PT is one of the most promising potential alternatives or adjuncts to antibiotics based on their ability to eradicate multi-drug resistant (MDR) bacterial infections [15,17]. However, despite the demonstrated efficacy of fully characterised phages against MDR infections administered on compassionate grounds, there are still many factors that require further investigation such as optimal pulmonary delivery mechanisms and the continued development of appropriate preclinical models [6].

## 2. Pulmonary Phage Therapy

Most applications of PT use a combination of phages mixed into a single preparation called a phage cocktail [15,18,19,20]. This requires multiple phages that are readily able to infect and lyse the target bacterium. However, if these do not exist within a current isolated bank of phages, then novel phages must be isolated and characterised, which can be time-consuming and laborious to complete on an ad hoc basis. The feasibility of using phage cocktails in treating bacterial respiratory infections this way to solve urgent needs is in question as this presents many challenges, including phage availability. One study that addresses this issue investigated the probabilities of isolating phage “on-demand” active against various MDR pathogens such as *Staphylococcus aureus*, *Escherichia coli*, *Klebsiella pneumoniae*, and *Pseudomonas aeruginosa* [21]. Their results indicated variable densities of infectious phage within their samples and those able to infect MRSA were particularly scarce [21]. Furthermore, individual phages demonstrated highly variable stability, which is consistent throughout the literature published so far [21,22,23] and has important consequences for generating the amounts of phage required for therapeutic application. 

While PT for bacterial infections has not yet made its way into standard care due to insufficient preclinical data, PT has been accessed for use on compassionate and investigational grounds with few reports of adverse events [16,24,25,26]. Many of these cases have been for patients with MDR or even pan-drug-resistant bacterial lung infections where all other therapeutic options for eradication had been exhausted [15,16]. The use of PT on compassionate grounds targets organisms that are prone to becoming drug resistant; the most frequent cases are for *S. aureus*, *P. aeruginosa*, and *E. coli* [25]. To date, pulmonary PT has been predominantly applied in cohorts of people that suffer from muco-obstructive lung diseases such as cystic fibrosis (CF), non-CF bronchiectasis, primary ciliary dyskinesia, and chronic obstructive pulmonary disease (COPD) [27]. Indeed, many compassionate uses of PT for lung infections are for CF sufferers who are at end-stage lung disease and have exhausted all other eradication options [15,16,20].

In all medical applications of PT, careful selection of phages is required to ensure both safety and efficacy. Attributes such as adsorption, replication, and distribution must be determined in addition to full genomic and morphological characterisation of each phage within a phage cocktail [12,28]. As the phage preparation practices and buffer systems in which phage are stored have an impact on the application of PT and patient safety, these must first be reviewed prior to medical applications [5,19]. Unfortunately, lag times between the current need for an approved treatment and the earliest foreseeable approvals for alternative antimicrobials are worrying, with newly approved therapies taking approximately 12 years to arise from preclinical studies in the USA [29]. This, in conjunction with the estimates of deaths attributable to resistant infections, ~700,000 per year already, have left us at an impasse [30]. 

### Pulmonary Delivery

Phages are diverse in nature and exhibit a range of unique biochemical and morphological features, which can influence their suitability to the airway environment [22]. Owing to this fact, phage stability at physiological temperatures and pH within delivery formulations must be known, as phage viability at the organ surface will dictate its lytic capabilities [31]. Whilst bactericidal activity is an important aspect, there remains several important considerations when using virulent phage to clear infections. One relates to the delivery method, which can be in formats such as nebulised liquid phage suspensions, inhalable dry powders, or hydrogels to transport phage directly to the organ surface [31,32]. Even though the use of hydrogels as a delivery method has produced promising results thus far, this method would be unsuitable to pulmonary delivery as it is designed for topical applications [31].

Delivery formulations of phage have provided both an efficacy and stability challenge for researchers and phage preparations in early clinical trials involving stabilising agents such as glycerol [18,19]. Nebulisation is widely utilised in respiratory medicine to deliver therapeutics to the deepest airway passages, using vibrating mesh, compressed air (jet nebulisation), or ultrasound to produce aerosols from liquid suspensions [33]. Nebulisation methods have been shown to reduce infectivity, measured by titre decrease, which correlated to morphological damage to the phages [33,34]. Furthermore, air-jet nebulisation was reported to damage the structural integrity of phages more than mesh nebulisation, and the extent of this damage was associated with tail length [34]. 

A study comparing the efficacy of two different delivery methods (intraperitoneal injection and aerosol inhalation) in an animal model of MDR *Burkholderia cepacia* complex (BCC) illustrated that aerosolised PT can be significantly more effective at treating respiratory infections [35]. This method was able to deliver phage at titres ~10^7^ directly into the respiratory tract using a jet-nebuliser and inhalation device; phage delivered this way caused statistically significant reductions in BCC in the lungs of mice, whereas mice injected intraperitoneally did not [34,35]. The stability of phage post-nebulisation is phage-dependent and demonstrates the need to evaluate in vitro propagation to high titres before moving onto more costly in vivo models.

An alternative to nebulisation is dry powder inhalation, a method that sprays dried phage into sugar-containing particles that are then stored as a dry powder [36,37]. The dry powder phage preparations produced this way have been assessed for stability and delivery efficacy over a range of conditions at 4 °C, with minor drops in viable titre after 12 months [37], suggesting it may be more advantageous than its liquid suspension counterpart. In addition to storage considerations, the measurements of aerosol performance also need to be assessed for pulmonary delivery. Chang et al. (2017) reported good phage aerosol performance as measured by the fine particle fraction (FPF) of their PEV20 phage formulation, far exceeding the FPF values of most commercial inhalers (20–30% FPF) [38]. Overall, phage delivery methods have been developed to the minimum standard required for compassionate use of PT in targeting pulmonary MDR infections. However, there remains significant potential to refine these processes to reduce variability in preparation delivery, and thus, improve the feasibility of clinical trials comparing PT to existing treatments. As organ-specific delivery methods continue to improve, appropriate in vitro assessments of aerosol performance should be performed following the isolation of novel phages to assess suitability for delivery.

## 3. Preclinical Data Generation

Preclinical data generation for PT follows a general trend from the isolation of phage, from environmental and/or clinical sources, through to characterisation in multiple aspects (Table 1) and then validation in animal models [21,39]. It has been stated in several publications that PT has not yet made its way into standard care due to the lack of these data and a lack of knowledge surrounding bacterial–host interactions [8,24]. For the translation of preclinical studies to clinical trials, sufficient preclinical data must be supplied to inform clinical use of investigational products. Such data include a combination of relevant in vitro data centred on measurements of infectivity and safety confirmations. These data must be generated using samples which have been produced in accordance with good manufacturing practices (GMP), as clinical administration should be accurately represented by the models used to inform the trial. Examples of this include a phage product, made according to GMP standards, designed to treat two infectious MDR pathogens, *S. aureus* and *P. aeruginosa* [40], and another phage product called AB-SA01, which also satisfied criteria for a phase 1 clinical trial [26,41]. The production of these products is heavily based on in-depth characterisations, such as genome sequencing required to confirm the absence of virulence and antibiotic resistance genes, morphological confirmation of the expected virion particles, and infectivity measurements against relevant and representative bacterial species [40,41]. 

### 3.1. In Vitro Models

In the context of PT to treat respiratory infections, developing appropriate in vitro models that can accurately recapitulate the human airway is vital in understanding how novel phages will interact with airway surface liquid, mucous and biofilm [46]. Many in vitro models of the lung epithelium thus far have used immortalised cell lines, including NCI-H441 and A549, which are human pulmonary epithelial cells [47]. A significant drawback of using these transformed or immortalised cells is their limited ability to truly generate the complex cellular stratification and physiology of the lung epithelium [48]. This, in conjunction with their inability to produce a phenotype marked by persistent inflammation and mucous production, mean they do not represent a chronically infected epithelial layer. Primary airway epithelial cells (pAECs) cultured at the air–liquid interface (ALI) [49] are better able to recapitulate these features of airway physiology by proper pseudo-stratification into multiple cell types with mucous production [49,50,51].

One concern for PT is that it may induce or exacerbate inflammation in the airways. To determine this, inflammatory biomarkers produced by the above models in response to phage can be measured [46]. The effects of phage on inflammatory immune cells such as the neutrophil, which is often a key driver of airway damage in obstructive pulmonary diseases [52], is now receiving greater attention. Anti-inflammatory effects have been suggested where significant reductions in C-reactive protein and white blood cell counts have been observed in patients treated with phage lysates [53]. However, this appears dependent upon whether phage preparations have been purified [54]. A review of phage safety and toxicity profiles has recently been conducted by Liu and colleagues [55]. The authors highlight shortfalls in reporting phage preparations, specifically their purity and composition, which may contain bacterial contaminants such as endotoxin [54,55] (a potent inducer of inflammation), and/or enterotoxins. Much of the literature to date supports the finding that phages do not incite inflammation in pulmonary tissues when adequately purified [46,56] and recent insights into host-phage reactions suggest phages exert anti-inflammatory effects via mechanisms unrelated to their antibacterial action [57].

To the authors’ knowledge, the only publication measuring the effects of phage on neutrophilic migration was conducted using Wild-type and hyper-inflamed *cftr* loss-of-function zebrafish embryo models [58]. Using these, it was determined that a four-phage cocktail had immunomodulatory effects via the downregulation of pro-inflammatory cytokines and reduced neutrophil migration in response to acute inflammatory induction by tailfin amputation [58]. Recently, the development of a transepithelial migration model has enabled researchers to measure polymorphonuclear neutrophilic migration through epithelial monolayers at ALI in response to a stimulus such as purified airway supernatants from CF patients [59]. Utilising this model to determine how phage exposure might affect the function of dynamically active neutrophils recruited into the airways may help to increase the amount of relevant preclinical data supporting the safety of PT for pulmonary infection. This could provide a significant improvement upon the previously mentioned zebrafish model due to the closer recapitulation of the airway environment and use of human pAECs.

Another issue with respect to studying phage in a preclinical setting is that respiratory bacterial infections are often associated with biofilm formation and for many bacterial pathogens, replicating this biofilm in vitro can be difficult [8,60]. Biofilms are complex structures that enable populations of bacteria, or multiple species of bacteria, to resist antibiotics at concentrations that would otherwise eradicate their planktonic counterparts [45]. Some phages have already shown promising results in removing biofilms (such as the *Sb-1 S. aureus* phage); however, in some cases, biofilms have been associated with enhanced resistance to phage infection and some may contain phage inactivating enzymes [45,60]. As this effect seems to be dependent on interactions between phage-resistant aspects of the biofilm and biofilm dispersal mechanisms of the phage, it is important to incorporate appropriate biofilm penetration assays into the characterisations of newly isolated phage [45,51]. 

Biofilm penetration and dispersal highlight another area of concern with antimicrobial agents, namely the downstream effects of bacterial lysis. For example, some antibiotics can induce a large release of bacterial cell wall components such as lipopolysaccharide (LPS) from Gram-negative bacteria that can result in septic shock [61]. There are data suggesting phages have an immunomodulatory rather than inflammatory effect; however, this remains pertinent, especially in situations where inflammation is a primary concern for the patient [58,62]. Concerns surrounding bacterial lysis could be addressed by using in vitro models to perform biofilm dispersal assays on pAECs in response to phage-mediated dispersal. The use of pAECs as a tool to study biofilm formation and dispersal of major airway pathogens upon coinfection with respiratory virus has been demonstrated [63]. However, biofilm models on pAEC have not yet been applied to PT, and biofilm penetration capacities are often measured on abiotic surfaces or using immortalised cell line models [64,65].

### 3.2. In Vivo Models

Following in vitro testing, the use of animal models is a necessary component in assessing the safety and efficacy of new therapeutics within a fully functional biological system. As mentioned previously, one safety concern with bactericidal agents, such as antibiotics and lytic phage, is the release of bacterial endotoxin upon cell lysis [61]. Fortunately, many of the results generated in vitro have been reflected in vivo thus far. For example, Dufour and colleagues reported that phage causing rapid bacterial lysis did not cause as much endotoxin release as various Beta-lactam antibiotics using an in vitro model [66]. Similarly, an acute pneumonia murine infection model showed that phage treatment was associated with lower levels of inflammation than antibiotics [67]. These results are also supported across other in vivo studies with different bacteria isolated, including MDR *P. aeruginosa* and *S. aureus*, common respiratory pathogens [68,69].

Existing in vivo models of PT have been typically designed to study phage efficacy in response to an acute infection in which animals are challenged with lethal doses of bacteria [8,35,70,71,72,73]. While providing a plethora of useful safety and efficacy data, PT is not usually used in this way in humans. As such, a model in which longer-term infection can be maintained and studied has been developed [74]. Fothergill et al. describe a mouse inhalation model in which *P. aeruginosa* is introduced intranasally and colonises the nasopharynx, followed by migration towards the lungs causing a lower respiratory tract infection over the course of 28 days. This enables comparison of *P. aeruginosa* isolated at both early and late stages of infection. Interestingly, their analysis of isolates taken at various timepoints indicated that the bacteria became resistant to tobramycin in the absence of antibiotic pressure [74]. The importance of this study towards pulmonary PT is evident as despite the phenotypic similarities between the original *P. aeruginosa* isolate and two isolates collected from the same mouse at day 21, a re-challenge experiment with these isolates confirmed that post-infective *P. aeruginosa* was far more adept at colonising the lower respiratory tract in vivo [74]. In comparison to previous studies utilising beads impregnated with bacteria to mimic a chronic infection, this model more accurately represents the natural development route of a persistent infection brought about by stable colonisation of the nasopharynx followed by migration to the lower respiratory tract after adequate adaptations have been acquired [74]. 

The delivery route in many of these models utilises intranasal administration which has provided support in favour of PT use by demonstrating bacterial reductions using both curative and preventative doses of phage [73,75]. Whilst easy to perform, this method is not without disadvantages, as a *lux* tagged strain of *P. aeruginosa* MR299 was visualised from 2–8 h post intranasal administration and found to be localised in the head and stomach of the mice as well as the lungs [75]. In acknowledgement of this, further studies have been performed using intratracheal administration of dry powder phage preparations in vivo that support the safety and efficacy of dry powdered formulations, which retain bactericidal activity (0.3-log_10_ titre drop) and demonstrate good aerosol performance in vitro (FPF 51.6%).

Though many animal models used thus far have demonstrated the safety and efficacy of PT, the majority of these incorporate a single bacterial species and a high dose of phage specific for treatment [67,68,71,73]. To date, there is a paucity of data on the efficacy of phage against polymicrobial infections, and the models developed thus far do not, to our knowledge, translate this polymicrobial aspect into their models. Despite the lack of polymicrobial contexts in preclinical data generation, PT has been used to clear a polymicrobial bone infection caused by *K. pneumoniae* and *A. baumannii* and, whilst successful, was administered in conjunction with the antibiotics Colistin and Meropenem [76]. The importance of incorporating polymicrobial aspects into in vivo models is required to further support the safety of PT within various infection scenarios. This is emphasised by publications indicating that PT could potentially have adverse consequences in scenarios where complex microbial communities exist, as is often the case in the lungs [77]. Furthermore, preclinical data generation for specific phage preparations must represent the clinical scenario in which it is used as accurately as possible. Expansion of models which enable establishment of LRTIs over a longer time period, representing chronic infections more accurately, should be a priority for pulmonary PT aimed at the usual clinical scenarios of patients whose case history has exhausted all other treatment options [15,16,74]. 

## 4. Clinical Trials Involving Phage for Respiratory Infections

There will always be caveats when using animal models to represent human conditions, but it is promising that human clinical trials of PT have reported no adverse events, aligning with results of in vivo animal models [8,26,78]. The small number of clinical trials conducted to date have demonstrated the safety and tolerability of phage preparations when administered intravenously or topically for otitis media, or wound sites [18,19,26]. Outside of the respiratory context, a phase 1 clinical trial demonstrated safe topical applications of phages to venous leg ulcers with no reports of adverse events [18]. In addition to this, another clinical trial (Trial registration: NCT03395769) was conducted in an Australian hospital which used intravenously administered phage as an adjunctive therapy [26], also with no adverse events reported. These phage preparations were designed according to GMP standards and, whilst promising for phage therapy in general, have not administered nebulised phages directly to the lungs [18,19,26,79]. Regardless of delivery route, general safety parameters have been established by these uses of PT; a more in-depth review of clinical trials and the regulatory hurdles they face is described by Furfaro and colleagues (2018) [24]. Unfortunately, the number of clinical trials that utilise phage for respiratory infections is few and many of these studies have not yet completed or published the findings (Table 2).

Clinical trials are necessary in demonstrating that safety and efficacy data generated in a preclinical laboratory environment are translated reliably into clinical practice. However, the disparity between the amount of preclinical data and actively recruiting clinical trials for PT is vast. This may be explained, in part, by the difficulties of producing or obtaining phage preparations made according to GMP standards as labs must be outfitted correctly and have the necessary accreditations [80]. These difficulties are compounded by the associated costs and time involved with obtaining GMP certified preparations. As such, in Western medicine, there has been no market authorisation or approval for phage products to treat human infections at the time of this review [41]. Though the regulatory aspects of PT lie outside the scope of this review, there has been significant progress in the regulated implementation of personalised phage therapeutics [80,81]. The magistral framework set in place by relevant health authorities has enabled the use of phage preparations prepared by a pharmacist and responsible clinician [80]. Bretaudeau and colleagues (2020) describe this approach as tailored to treat individuals who have exhausted all other treatment options [80]. However, this framework has enabled the use of magistral preparations in clinical trials regulated on a national level, which has been recently performed by a multidisciplinary phage task force (*The PHAGEFORCE Study Protocol*) [82].

## 5. Resistance

Resistance to phage is undesirable yet unavoidable with multiple mechanisms de-scribed in bacteria. As with antibiotics, the emergence of bacterial resistance to phage is observable using standard culturing procedures in the lab [78]. This has led to a number of publications that address this area of concern, one of which reviewed resistance development across numerous animal models of infection and treatment with PT [79]. In recent years, a number of novel resistance mechanisms have been described such as the DISARM and BREX systems [78,80]. However, the emergence of resistance in vitro is not always reflected in vivo. This is thought to be because the more common mutations conferring resistance to the phage (the attachment interference resistance mechanism) are for the cell surface molecules that are also required for infectivity within the host [78]. Phage training is a method that may be able to aid in delaying bacterial resistance by producing phage derivatives [45,81]. This works by allowing known lytic phage to evolve and overcome the defences of a partially resistant bacterial strain in vitro [45,81]. These methods have been published and derivates of known phage, active against *S. aureus*, have been used to prevent biofilm formation of *S. aureus* and reduce the density of previously established biofilms in vitro [45].

Fortunately, findings from the majority of these studies indicate that phage resistance has an attenuative effect on bacterial virulence [83], generally thought to be due to fitness costs imposed on bacteria with mutations or adaptive defence mechanisms such as CRISPR [84]. Due to the personalised nature of phage preparations used in clinical trials thus far, their justification has taken this concern into account by quantifying the rate of resistance development; this was carried out to inform the clinical trial for AB-SA01 phage product [26,41]. These findings do not necessarily mean that PT will not face issues with resistance in the future, as short-term and long-term resistance to phages may use disparate mechanisms and bacterial isolates may enter a coevolution cycle in which both populations may expand in parallel [84]. To address this concern, proactive monitoring and characterisation of pathogenic bacteria and the antiviral mechanisms that they harbor is required to sustain the accuracy of identifying effective phage within a continuously evolving phage bank.

Some bacterial species will require more stringent characterisation than others, as the abundance of phage resistance mechanisms and the amount of interference from prophage are highly variable between isolates [85]. Prophages are phages that may not obligately follow the virulent cycle of replication and may integrate into their bacterial hosts’ genome [86]. It is generally recommended that production strains that are free from prophage be used to amplify phage products for therapeutic application; however, these can be difficult to obtain [86]. In addition to this, basic characterisations such as host-range assays can become increasingly difficult to assess considering the hierarchical nature of resistance mechanisms found in some bacterial species [85]. To fully understand these complex reactions, more clinically relevant bacterial and phage isolates must be characterised regardless of their lifecycle and therapeutic potential. Further, the associations between prophages, resistance, and their propensity to contaminate therapeutic products have led researchers to realise the importance of readily available production strains for certain bacterial species and the characterisation of prophage within them [86].

## 6. Concluding Remarks

The development of PT for lung infections has come a long way, with successful use in treating infections on a compassionate basis [15,16,25], and presents a significant opportunity for more conventional applications. For pulmonary PT to become a standard treatment option, the focus in preclinical studies should be on expanding in vivo models to better recapitulate chronic infections of the several bacterial species common to MDR respiratory disease and measuring their phage–biofilm interactions on airway epithelial cell layers rather than abiotic surfaces. Preclinical data for pulmonary PT might also benefit from the inclusion of neutrophil migration models in response to phage, or phage-derived products, as neutrophilic inflammation is an acute concern in respiratory disease and results in further lung damage or respiratory failure. These investigations should spur the increases in clinical trials necessary for PT to become a standard treatment option for treatment of MDR lung infections and perhaps even prevention of MDR in people living with long-term chronic respiratory diseases.

## Figures and Tables

**Table 1:** List of typical phage characterisations and the data obtained.

Characterisation	Data Obtained	References
Infectivity: Host-range and specificity	Host-range and specificity assays determine the ability for a single phage to infect different bacterial strains, species, and genera. However, to determine how well a phage can infect any given strain of bacteria, an efficiency of plating assay must be performed by spotting a serial dilution of phage onto lawns of bacterial culture. Many studies utilise standard microbiological plating techniques to do these, such as the spot test on double agar overlays inoculated with the target bacteria.	[21,28,39,42,43]
Morphological analysis	Homogenous phage particles are prepared, via staining of high titre phage lysates, for observation via transmission electron microscopy. From this, the head size and shape, as well as tail length, are determined.	[28,39,42,43]
Adsorption assay	Adsorption assays are used to determine how fast a phage attaches to its target bacterium and the percentage of phage within a sample that can attach to the target bacterium at a given multiplicity of infection (MOI).	[28,42]
One-step growth curve	Latency period is the period between adsorption of phage to the target bacteria and the first burst as indicated by the rise in phage titre within a sample. The burst size of a phage refers to how many virions are released per infected bacterial cell and both are typically calculated using the one-step growth curve.	[28,42,43]
Kill-curves/Lysis profiles	These are bacteriolytic activity tests which determine a phages lytic capability in vitro by infecting early exponential phase bacteria at various MOIs and comparing with a non-infected control. Bacterial cell density is typically measured via spectrophotometry at an optical density of 600 nm.	[28,42,43]
Stability/Sensitivity	The stability of phages at various temperatures and pH ranges is important to determine how fast phages will degrade under different storage or relevant test conditions, such as body temperature.	[21,28,39,42]
Genomic analysis	Genomic analysis can be carried out in various ways such as restriction enzyme analysis and whole genome sequencing of genomic DNA (gDNA) isolated from phage. Restriction enzyme analysis is performed using restriction endonucleases to cleave the gDNA, followed by gel electrophoresis to visualise restriction patterns and estimate the size of the genome. Whole genome sequencing gives the ability to analyse and annotate the full genome of a phage, perform whole genome-based phylogeny, and search for lysogeny-associated genes such as integrases.	[28,42,43]
Biofilm susceptibility	Biofilm-forming bacteria are grown in specialised growth media to promote the formation of biofilm on abiotic surfaces. Typically, this is carried out over 24 and 48 h, as the age of biofilms has an effect on bacterial susceptibility to a number of agents including both phage and antibiotics. In addition to biofilm clearance, prevention has also been measured on abiotic surfaces.	[42,44,45]

**Table 2:** Clinical trials involving the use of PT for respiratory infections. Clinical trial ID refers to the ClinicalTrials.gov identifier code. Included are only clinical trials that have the term “Bacteriophage” used as a key word and those that are for the treatment of respiratory infection.

Study Title	Clinical Trial ID	Primary and Secondary Outcome Measurements	Phage Treatment	Pathogen Details	Status at Time of Writing
Bacteriophage Therapy in Acute Tonsillitis.	NCT04682964	Clinical observation of sick children. Manifestation of clinical symptoms, observation of the nasopharynx, and parental report. Experimental clinical stage: Bacteriophage therapy, efficacy will be studied based on immunological assay, cellular and humoral immunity via ELISA.	Pyobacteriophage complex liquid (PCL) administered via nebuliser inhalation.	Not available	Active, not recruiting.
Cystic Fibrosis bacteriophage Study at Yale (CYPHY): A Single-site, Randomized, Double-blind, Placebo-controlled Study of Bacteriophage Therapy YPT-01 for *Pseudomonas aeruginosa* Infections in Adults With Cystic Fibrosis.	NCT04684641	Primary: Reduction in sputum bacterial culture. Secondary: Change in lung function based on FEV at multiple stages. Change in rate of pulmonary exacerbations. Change in the rate of hospitalisation. Change in the rate of acute antibiotic usage. Patients’ quality of life.	Yale Phage Therapy (YPT)-01	*P. aeruginosa*	Recruiting.
A Phase 1b/2a, Multi-Center, Double-Blind, Randomized, Placebo-Controlled, Single and Multiple Ascending Dose Study to Evaluate the Safety and Tolerability of AP-PA02 Multi-Phage Therapeutic Candidate for Inhalation in Subjects With Cystic Fibrosis and Chronic Pulmonary *Pseudomonas aeruginosa* (Pa) Infection.	NCT04596319	Primary: Incidence of treatment emergent adverse events for safety and tolerability of single and multiple doses. Secondary: Explore *P. aeruginosa* recovery from sputum following multiple doses of AP-PA02 as measured by change in CFU/g of sputum.	AP-PA02 phage product administered via inhalation.	*P. aeruginosa*	Recruiting.

## Data Availability

Not applicable.

## References

[1] Azoulay E., Russell L., Van De Louw A., Metaxa V., Bauer P., Povoa P., Montero J.G., Loeches I.M., Mehta S., the Nine-i Investigators (2020). Diagnosis of severe respiratory infections in immunocompromised patients. Intensiv. Care Med..

[2] Pragman A.A., Berger J.P., Williams B.J. (2016). Understanding Persistent Bacterial Lung Infections. Clin. Pulm. Med..

[3] Orgeur M., Brosch R. (2018). Evolution of virulence in the Mycobacterium tuberculosis complex. Curr. Opin. Microbiol..

[4] Troeger C., Forouzanfar M., Rao P.C., Khalil I., Brown A., Swartz S., Fullman N., Mosser J., Thompson R.L., Reiner R.C. (2017). Estimates of the global, regional, and national morbidity, mortality, and aetiologies of lower respiratory tract infections in 195 countries: A systematic analysis for the Global Burden of Disease Study 2015. Lancet Infect. Dis..

[5] Chang R.Y.K., Wallin M., Lin Y., Leung S., Wang H., Morales S., Chan H.-K. (2018). Phage therapy for respiratory infections. Adv. Drug Deliv. Rev..

[6] Cassini A., Högberg L.D., Plachouras D., Quattrocchi A., Hoxha A., Simonsen G.S., Colomb-Cotinat M., E Kretzschmar M., Devleesschauwer B., Cecchini M. (2018). Attributable deaths and disability-adjusted life-years caused by infections with antibiotic-resistant bacteria in the EU and the European Economic Area in 2015: A population-level modelling analysis. Lancet Infect. Dis..

[7] Principi N., Silvestri E., Esposito S. (2019). Advantages and Limitations of Bacteriophages for the Treatment of Bacterial Infections. Front. Pharmacol..

[8] Melo L.D.R., Oliveira H., Pires D.P., Dabrowska K., Azeredo J. (2020). Phage therapy efficacy: A review of the last 10 years of preclinical studies. Crit. Rev. Microbiol..

[9] Hoe S., Semler D.D., Goudie A.D., Lynch K.H., Matinkhoo S., Finlay W.H., Dennis J., Vehring R. (2013). Respirable Bacteriophages for the Treatment of Bacterial Lung Infections. J. Aerosol Med. Pulm. Drug Deliv..

[10] Wittebole X., De Roock S., Opal S.M. (2013). A historical overview of bacteriophage therapy as an alternative to antibiotics for the treatment of bacterial pathogens. Virulence.

[11] Sulakvelidze A., Alavidze Z., Morris J.G. (2001). Bacteriophage Therapy. Antimicrob. Agents Chemother..

[12] Wienhold S.-M., Lienau J., Witzenrath M. (2019). Towards Inhaled Phage Therapy in Western Europe. Viruses.

[13] Bodner K., Melkonian A.L., Covert M.W. (2020). The Enemy of My Enemy: New Insights Regarding Bacteriophage–Mammalian Cell Interactions. Trends Microbiol..

[14] Górski A., Międzybrodzki R., Żaczek M., Borysowski J. (2020). Phages in the fight against COVID-19?. Futur. Microbiol..

[15] Law N., Logan C., Yung G., Furr C.-L.L., Lehman S.M., Morales S., Rosas F., Gaidamaka A., Bilinsky I., Grint P. (2019). Successful adjunctive use of bacteriophage therapy for treatment of multidrug-resistant Pseudomonas aeruginosa infection in a cystic fibrosis patient. Infection.

[16] Gainey A.B., Burch A., Brownstein M.J., Brown D.E., Ms J.F., Bs B.H., Biswas B., Bivens B.N., Malagon F., Daniels R. (2020). Combining bacteriophages with cefiderocol and meropenem/vaborbactam to treat a pan-drug resistant Achromobacter species infection in a pediatric cystic fibrosis patient. Pediatr. Pulmonol..

[17] Trend S., Fonceca A.M., Ditcham W.G., Kicic A., Cf A. (2017). The potential of phage therapy in cystic fibrosis: Essential human-bacterial-phage interactions and delivery considerations for use in Pseudomonas aeruginosa-infected airways. J. Cyst. Fibros..

[18] Rhoads D., Wolcott R., Kuskowski M., Wolcott B., Ward L., Sulakvelidze A. (2009). Bacteriophage therapy of venous leg ulcers in humans: Results of a phase I safety trial. J. Wound Care.

[19] Wright A., Hawkins C., Änggård E., Harper D. (2009). A controlled clinical trial of a therapeutic bacteriophage preparation in chronic otitis due to antibiotic-resistantPseudomonas aeruginosa; a preliminary report of efficacy. Clin. Otolaryngol..

[20] Kutateladze M., Adamia R. (2008). Phage therapy experience at the Eliava Institute. Médecine Mal. Infect..

[21] Emattila S., Eruotsalainen P., Ejalasvuori M. (2015). On-Demand Isolation of Bacteriophages Against Drug-Resistant Bacteria for Personalized Phage Therapy. Front. Microbiol..

[22] Jurczak-Kurek A., Gąsior T., Nejman-Faleńczyk B., Bloch S., Dydecka A., Topka G., Necel A., Jakubowska-Deredas M., Narajczyk M., Richert M. (2016). Biodiversity of bacteriophages: Morphological and biological properties of a large group of phages isolated from urban sewage. Sci. Rep..

[23] González-Menéndez E., Fernández L., Gutiérrez D., Rodríguez A., Martínez B., García P. (2018). Comparative analysis of different preservation techniques for the storage of Staphylococcus phages aimed for the industrial development of phage-based antimicrobial products. PLoS ONE.

[24] Furfaro L.L., Payne M.S., Chang B.J. (2018). Bacteriophage Therapy: Clinical Trials and Regulatory Hurdles. Front. Cell. Infect. Microbiol..

[25] McCallin S., Sacher J., Zheng J., Chan B.K. (2019). Current State of Compassionate Phage Therapy. Viruses.

[26] Fabijan A.P., Lin R.C.Y., Ho J., Maddocks S., Ben Zakour N.L., Iredell J.R., Khalid A., Venturini C., Chard R., Morales S. (2020). Safety of bacteriophage therapy in severe Staphylococcus aureus infection. Nat. Microbiol..

[27] Boucher R.C. (2019). Muco-Obstructive Lung Diseases. New Engl. J. Med..

[28] Łubowska N., Grygorcewicz B., Kosznik-Kwaśnicka K., Zauszkiewicz-Pawlak A., Węgrzyn A., Dołęgowska B., Piechowicz L. (2019). Characterization of the Three New Kayviruses and Their Lytic Activity Against Multidrug-Resistant Staphylococcus aureus. Microorganisms.

[29] Van Norman G.A. (2016). Drugs, Devices, and the FDA: Part 1. JACC: Basic Transl. Sci..

[30] O’Neill J. (2014). Tackling a Crisis for the Health and Wealth of Nations.

[31] Kim H., Chang R., Morales S., Chan H.-K. (2021). Bacteriophage-Delivering Hydrogels: Current Progress in Combating Antibiotic Resistant Bacterial Infection. Antibiotics.

[32] Chang R.Y.K., Wallin M., Kutter E., Morales S., Britton W., Li J., Chan H.-K. (2019). Storage stability of inhalable phage powders containing lactose at ambient conditions. Int. J. Pharm..

[33] Astudillo A., Leung S.S.Y., Kutter E., Morales S., Chan H.-K. (2018). Nebulization effects on structural stability of bacteriophage PEV 44. Eur. J. Pharm. Biopharm..

[34] Leung S., Carrigy N.B., Vehring R., Finlay W.H., Morales S., Carter E., Britton W.J., Kutter E., Chan H.-K. (2018). Jet nebulization of bacteriophages with different tail morphologies–Structural effects. Int. J. Pharm..

[35] Semler D.D., Goudie A.D., Finlay W.H., Dennis J.J. (2014). Aerosol Phage Therapy Efficacy in Burkholderia cepacia Complex Respiratory Infections. Antimicrob. Agents Chemother..

[36] Chang R.Y.K., Wong J., Mathai A., Morales S., Kutter E., Britton W., Li J., Chan H.-K. (2017). Production of highly stable spray dried phage formulations for treatment of Pseudomonas aeruginosa lung infection. Eur. J. Pharm. Biopharm..

[37] Leung S., Parumasivan T., Gao F.G., Carter E., Carrigy N.B., Vehring R., Finlay W.H., Morales S., Britton W.J., Kutter E. (2017). Effects of storage conditions on the stability of spray dried, inhalable bacteriophage powders. Int. J. Pharm..

[38] Chang R.Y.K., Chen K., Wang J., Wallin M., Britton W., Morales S., Kutter E., Li J., Chan H.-K. (2018). Proof-of-Principle Study in a Murine Lung Infection Model of Antipseudomonal Activity of Phage PEV20 in a Dry-Powder Formulation. Antimicrob. Agents Chemother..

[39] Abatángelo V., Bacci N.P., Boncompain C.A., Amadio A.F., Carrasco S., Suárez C.A., Morbidoni H.R. (2017). Broad-range lytic bacteriophages that kill Staphylococcus aureus local field strains. PLoS ONE.

[40] Merabishvili M., Pirnay J.-P., Verbeken G., Chanishvili N., Tediashvili M., Lashkhi N., Glonti T., Krylov V., Mast J., Van Parys L. (2009). Quality-Controlled Small-Scale Production of a Well-Defined Bacteriophage Cocktail for Use in Human Clinical Trials. PLoS ONE.

[41] Lehman S.M., Mearns G., Rankin D., Cole R.A., Smrekar F., Branston S.D., Morales S. (2019). Design and Preclinical Development of a Phage Product for the Treatment of Antibiotic-Resistant Staphylococcus aureus Infections. Viruses.

[42] Jamal M., Hussain T., Das C.R., Andleeb S. (2015). Characterization of Siphoviridae phage Z and studying its efficacy against multidrug-resistant Klebsiella pneumoniae planktonic cells and biofilm. J. Med Microbiol..

[43] Han J.E., Kim J.H., Hwang S.Y., Choresca C.H., Shin S.P., Jun J.W., Chai J.Y., Park Y.H., Park S.C. (2013). Isolation and characterization of a Myoviridae bacteriophage against Staphylococcus aureus isolated from dairy cows with mastitis. Res. Veter- Sci..

[44] Kelly D., McAuliffe O., Ross R., Coffey A. (2012). Prevention of Staphylococcus aureus biofilm formation and reduction in established biofilm density using a combination of phage K and modified derivatives. Lett. Appl. Microbiol..

[45] Ferriol-González C., Domingo-Calap P. (2020). Phages for Biofilm Removal. Antibiotics.

[46] Trend S., Chang B.J., O’Dea M., Stick S., Kicic A., WAERP, AusREC, AREST CF (2018). Use of a Primary Epithelial Cell Screening Tool to Investigate Phage Therapy in Cystic Fibrosis. Front. Pharmacol..

[47] Ren H., Birch N.P., Suresh V. (2016). An Optimised Human Cell Culture Model for Alveolar Epithelial Transport. PLoS ONE.

[48] Garratt L.W., Sutanto E.N., Foo C.J., Ling K.M., Looi K., Kicic-Starcevich E., Iosifidis T., Martinovich K.M., Lannigan F.J., Stick S.M. (2014). Determinants of culture success in an airway epithelium sampling program of young children with cystic fibrosis. Exp. Lung Res..

[49] Martinovich K., Iosifidis T., Buckley A.G., Looi K., Ling K.-M., Sutanto E.N., Kicic-Starcevich E., Garratt L.W., Shaw N.C., Montgomery S. (2017). Conditionally reprogrammed primary airway epithelial cells maintain morphology, lineage and disease specific functional characteristics. Sci. Rep..

[50] Looi K., Troy N.M., Garratt L.W., Iosifidis T., Bosco A., Buckley A.G., Ling K.-M., Martinovich K., Kicic-Starcevich E., Shaw N.C. (2016). Effect of human rhinovirus infection on airway epithelium tight junction protein disassembly and transepithelial permeability. Exp. Lung Res..

[51] Ng R.N., Tai A.S., Chang B.J., Stick S.M., Kicic A. (2021). Overcoming Challenges to Make Bacteriophage Therapy Standard Clinical Treatment Practice for Cystic Fibrosis. Front. Microbiol..

[52] Elborn J.S. (2016). Cystic fibrosis. Lancet.

[53] Międzybrodzki R., Fortuna W., Weber-Dąbrowska B., Gorski A. (2009). A retrospective analysis of changes in inflammatory markers in patients treated with bacterial viruses. Clin. Exp. Med..

[54] Hietala V., Horsma-Heikkinen J., Carron A., Skurnik M., Kiljunen S. (2019). The Removal of Endo- and Enterotoxins From Bacteriophage Preparations. Front. Microbiol..

[55] Liu D., Van Belleghem J., de Vries C., Burgener E., Chen Q., Manasherob R., Aronson J., Amanatullah D., Tamma P., Suh G. (2021). The Safety and Toxicity of Phage Therapy: A Review of Animal and Clinical Studies. Viruses.

[56] Huh H., Wong S., Jean J.S., Slavcev R. (2019). Bacteriophage interactions with mammalian tissue: Therapeutic applications. Adv. Drug Deliv. Rev..

[57] Górski A., Dąbrowska K., Międzybrodzki R., Weber-Dąbrowska B., Łusiak-Szelachowska M., Jończyk-Matysiak E., Borysowski J. (2017). Phages and immunomodulation. Futur. Microbiol..

[58] Cafora M., Brix A., Forti F., Loberto N., Aureli M., Briani F., Pistocchi A. (2020). Phages as immunomodulators and their promising use as anti-inflammatory agents in a cftr loss-of-function zebrafish model. J. Cyst. Fibros..

[59] Forrest O.A., Ingersoll S.A., Preininger M., Laval J., Limoli D., Brown M.R., Lee F.E., Bedi B., Sadikot R.T., Goldberg J.B. (2018). Frontline Science: Pathological conditioning of human neutrophils recruited to the airway milieu in cystic fibrosis. J. Leukoc. Biol..

[60] Pires D.P., Melo L., Boas D.V., Sillankorva S., Azeredo J. (2017). Phage therapy as an alternative or complementary strategy to prevent and control biofilm-related infections. Curr. Opin. Microbiol..

[61] Lepper P., Held T., Schneider E., Bölke E., Gerlach H., Trautmann M. (2002). Clinical implications of antibiotic-induced endotoxin release in septic shock. Intensiv. Care Med..

[62] Górski A., Jończyk-Matysiak E., Łusiak-Szelachowska M., Międzybrodzki R., Weber-Dąbrowska B., Borysowski J. (2017). The Potential of Phage Therapy in Sepsis. Front. Immunol..

[63] Kiedrowski M.R., Gaston J.R., Kocak B.R., Coburn S.L., Lee S., Pilewski J.M., Myerburg M.M., Bomberger J.M. (2018). Staphylococcus aureus Biofilm Growth on Cystic Fibrosis Airway Epithelial Cells Is Enhanced during Respiratory Syncytial Virus Coinfection. mSphere.

[64] Hosseinidoust Z., Tufenkji N., Van De Ven T.G. (2013). Formation of biofilms under phage predation: Considerations concerning a biofilm increase. Biofouling.

[65] Chang R.Y.K., Das T., Manos J., Kutter E., Morales S., Chan H.-K. (2019). Bacteriophage PEV20 and Ciprofloxacin Combination Treatment Enhances Removal of Pseudomonas aeruginosa Biofilm Isolated from Cystic Fibrosis and Wound Patients. AAPS J..

[66] Dufour N., Delattre R., Ricard J.-D., Debarbieux L. (2017). The Lysis of Pathogenic Escherichia coli by Bacteriophages Releases Less Endotoxin Than by β-Lactams. Clin. Infect. Dis..

[67] Dufour N., Delattre R., Chevallereau A., Ricard J.D., Debarbieux L. (2019). Phage therapy of pneumonia is not associated with an overstimulation of the inflammatory response compared to antibiotic treatment in mice. Antimicrob. Agents Chemother..

[68] Jeon J., Yong D. (2019). Two Novel Bacteriophages Improve Survival in Galleria mellonella Infection and Mouse Acute Pneumonia Models Infected with Extensively Drug-Resistant Pseudomonas aeruginosa. Appl. Environ. Microbiol..

[69] Wang Z., Zheng P., Ji W., Fu Q., Wang H., Yan Y., Sun J. (2016). SLPW: A Virulent Bacteriophage Targeting Methicillin-Resistant Staphylococcus aureus In vitro and In vivo. Front. Microbiol..

[70] Prazak J., Iten M., Cameron D., Save J., Grandgirard D., Resch G., Goepfert C., Leib S.L., Takala J., Jakob S.M. (2019). Bacteriophages Improve Outcomes in Experimental Staphylococcus aureus Ventilator-associated Pneumonia. Am. J. Respir. Crit. Care Med..

[71] Huff W., Huff G., Rath N., Balog J., Donoghue A. (2003). Evaluation of aerosol spray and intramuscular injection of bacteriophage to treat an Escherichia coli respiratory infection. Poult. Sci..

[72] Carmody L.A., Gill J., Summer E.J., Sajjan U.S., Gonzalez C.F., Young R.F., Lipuma J.J. (2010). Efficacy of Bacteriophage Therapy in a Model ofBurkholderia cenocepaciaPulmonary Infection. J. Infect. Dis..

[73] Morello E., Saussereau E., Maura D., Huerre M., Touqui L., Debarbieux L. (2011). Pulmonary Bacteriophage Therapy on Pseudomonas aeruginosa Cystic Fibrosis Strains: First Steps Towards Treatment and Prevention. PLoS ONE.

[74] Fothergill J., Neill D., Loman N., Winstanley C., Kadioglu A. (2014). Pseudomonas aeruginosa adaptation in the nasopharyngeal reservoir leads to migration and persistence in the lungs. Nat. Commun..

[75] Alemayehu D., Casey P.G., McAuliffe O., Guinane C.M., Martin J.G., Shanahan F., Coffey A., Ross R., Hill C. (2012). Bacteriophages ϕMR299-2 and ϕNH-4 Can Eliminate Pseudomonas aeruginosa in the Murine Lung and on Cystic Fibrosis Lung Airway Cells. mBio.

[76] Nir-Paz R., Gelman D., Khouri A., Sisson B.M., Fackler J., Alkalay-Oren S., Khalifa L., Rimon A., Yerushalmy O., Bader R. (2019). Successful Treatment of Antibiotic-resistant, Poly-microbial Bone Infection With Bacteriophages and Antibiotics Combination. Clin. Infect. Dis..

[77] Secor P.R., Sass G., Nazik H., Stevens D.A. (2017). Effect of acute predation with bacteriophage on intermicrobial aggression by Pseudomonas aeruginosa. PLoS ONE.

[78] Park K., Cha K., Myung H. (2014). Observation of inflammatory responses in mice orally fed with bacteriophage T7. J. Appl. Microbiol..

[79] Jault P., Leclerc T., Jennes S., Pirnay J.P., Que Y.-A., Resch G., Rousseau A.-F., Ravat F., Carsin H., Le Floch R. (2018). Efficacy and tolerability of a cocktail of bacteriophages to treat burn wounds infected by Pseudomonas aeruginosa (PhagoBurn): A randomised, controlled, double-blind phase 1/2 trial. Lancet Infect. Dis..

[80] Bretaudeau L., Tremblais K., Aubrit F., Meichenin M., Arnaud I. (2020). Good Manufacturing Practice (GMP) Compliance for Phage Therapy Medicinal Products. Front. Microbiol..

[81] Pirnay J.P., Verbeken G., Ceyssens P.J., Huys I., De Vos D., Ameloot C., Fauconnier A. (2018). The Magistral Phage. Viruses.

[82] Onsea J., Uyttebroek S., Chen B., Wagemans J., Lood C., Van Gerven L., Spriet I., Devolder D., Debaveye Y., Depypere M. (2021). Bacteriophage Therapy for Difficult-to-Treat Infections: The Implementation of a Multidisciplinary Phage Task Force (*The PHAGEFORCE Study Protocol*). Viruses.

[83] Oechslin F. (2018). Resistance Development to Bacteriophages Occurring during Bacteriophage Therapy. Viruses.

[84] Torres-Barceló C. (2018). Phage Therapy Faces Evolutionary Challenges. Viruses.

[85] Moller A.G., Lindsay J., Read T.D. (2019). Determinants of Phage Host Range in Staphylococcus Species. Appl. Environ. Microbiol..

[86] Rohde C., Resch G., Pirnay J.-P., Blasdel B.G., Debarbieux L., Gelman D., Górski A., Hazan R., Huys I., Kakabadze E. (2018). Expert Opinion on Three Phage Therapy Related Topics: Bacterial Phage Resistance, Phage Training and Prophages in Bacterial Production Strains. Viruses.

